# Supplementary material for: Trypanosomatid research in Brazil: a systematic analysis of regional and temporal trends
Source: Mem Inst Oswaldo Cruz. 2026 Jan 12;120:e250098. doi: 10.1590/0074-02760250098 (PMC12795327; doi:10.1590/0074-02760250098)
Supplement: Supplementary material [file 1678-8060-mioc-120-e250098-s.pdf]

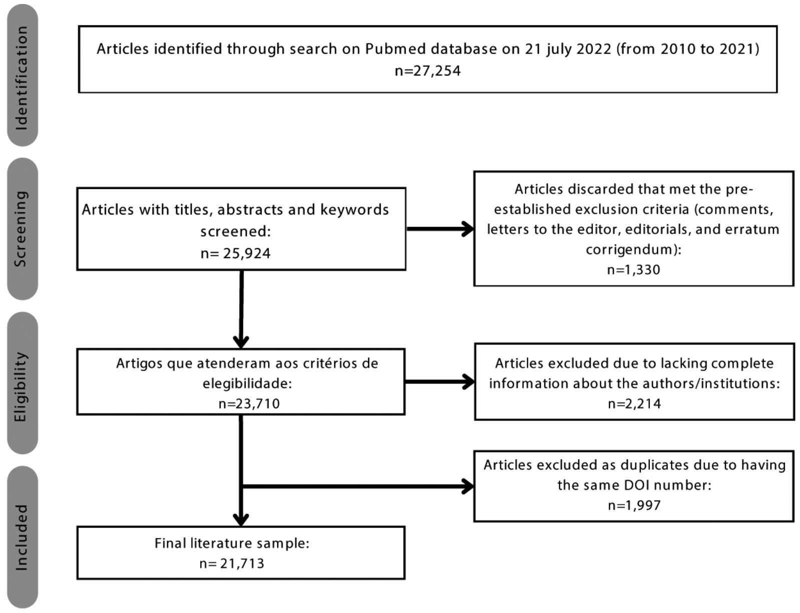

Fig. 1: flow diagram of the selection process.

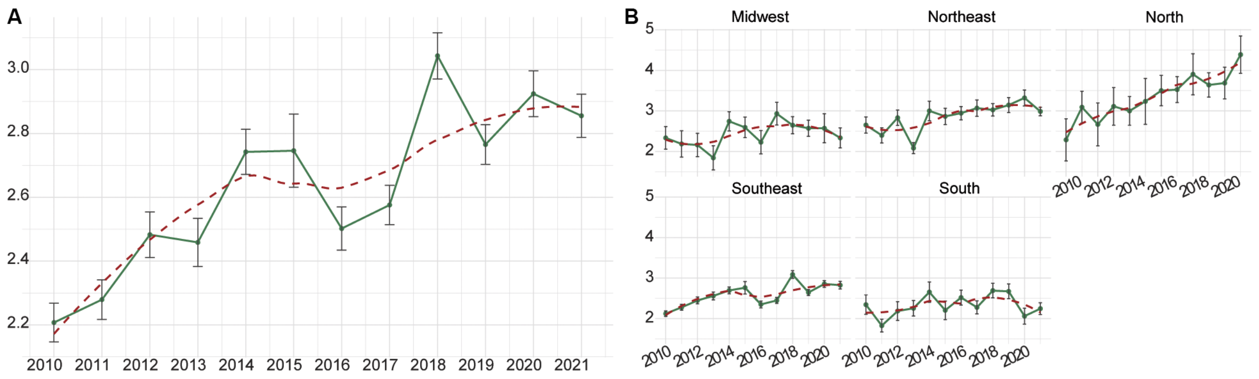

Fig. 2: evolution of the average number of author affiliations per article in Brazil (2010-2021). (A) Average number of author affiliations per scientific article in Brazil. The x-axis represents the years (from 2010 to 2021) and the y-axis, the average number of affiliations. The solid green line indicates the average with the standard deviation, while the dashed red line represents the trend over the period. (B) Average number of author affiliations per article, segmented by the five geographical regions of Brazil: Midwest, Northeast, North, Southeast, and South. Each subgraph details the evolution of the average number of affiliations per year in its respective region. The solid green line represents the mean with the standard deviation (SD), while the dashed red line indicates the trend for each region.
